# Screening of HHEX Mutations in Chinese Children with Thyroid Dysgenesis

**DOI:** 10.4274/jcrpe.2456

**Published:** 2016-03-01

**Authors:** Shiguo Liu, Jian Chai, Guohua Zheng, Huichao Li, Deguo Lu, Yinlin Ge

**Affiliations:** 1 The Affiliated Hospital of Qingdao University, Prenatal Diagnosis Center, Qingdao, China; 2 The Affiliated Hospital of Qingdao University, Genetic Laboratory, Qingdao, China; 3 Qingdao University Faculty of Medicine, Department of Biochemistry and Molecular Biology, Qingdao, China; 4 Weifang Maternal and Children Health’s Hospital, Weifang, China; 5 The Affiliated Hospital of Qingdao University, Department of Thyroid Surgery, Qingdao, China; 6 Linyi People’s Hospital, Department of Clinical Laboratory, Shandong, China; 7 These authors contributed equally to this work.

**Keywords:** congenital hypothyroidism, thyroid dysgenesis, HHEX, mutation

## Abstract

**Objective:**

Congenital hypothyroidism (CH) is a frequent neonatal endocrine disease with an incidence of about 1:2500 worldwide. Although thyroid dysgenesis (TD) is the most frequent cause of CH cases, its pathogenesis remains unclear. The aim of this study was to screen the hematopoietically-expressedhomeobox gene (HHEX) mutations in Chinese children with TD.

**Methods:**

Genomic deoxyribonucleic acid was extracted from peripheral blood leukocytes in 234 TD patients from Shandong Province. Mutations in all exons and nearby introns of HHEX were analyzed by direct sequencing after polymerase chain reaction amplification.

**Results:**

Sequencing analysis of HHEX indicated that no causative mutations were present in the coding regionof the TD patients. However, a genetic variant (IVS2+ 127 G/T, 10.26%) was observed in the intron 2 in HHEX.

**Conclusion:**

Our results indicate that the frequency of HHEX mutation is very low and may not be the main causative factor in Chinese TD patients. However, these results need to be replicated using larger datasets collected from different populations.

**WHAT IS ALREADY KNOWN ON THIS TOPIC?**

HHEX knock-out mouse strongly suggested that HHEX has no role in thyroid specification but is required to maintain Tif-1, Pax8, and Tif-2 expression in the developing thyroid.

**WHAT THIS STUDY ADDS?**

The frequency of HHEX mutation is very low and may not be the main causative factor in Chinese thyroid dysgenesis patients.

## INTRODUCTION

Congenital hypothyroidism (CH) is the most frequent endocrine metabolic disease in infancy and affects about 1/2500 newborns (1). This disease, if left untreated, can seriously affect the child’s physical and mental growth. CH was, until the introduction of the newborn screening program, one of the most important causes of mental retardation. With the exception of rare cases of central hypothyroidism, CH is characterized by elevated serum thyroid stimulating hormone (TSH) levels resulting from reduced thyroid hormone levels. In nearly 15% of cases, CH is caused by inborn errors of thyroid hormones biosynthesis. The term dyshormonogenesis is generally used for this condition, which is associated with goiter and shows classical Mendelian recessive inheritance. Dyshormonogenesis is often caused by mutations in the genes involved in the synthesis of thyroid hormone, such asiodotyrosinedeiodinase (IYD), dual oxidase 2 (DUOXA2), DUOX maturation factor 2 (DUOXA2), thyroglobulin (TG), thyroperoxidase (TPO), sodium/iodide symporter (NIS), and pendrin (PDS) (2). In the remaining 85%, casesare grouped under the term thyroid dysgenesis (TD) due to defects in thyroid gland development, which contains agenesis (35-40%), ectopy (30-45%), and hypoplasia (5%) (3). Some studies have been reported that some genes, such as paired box transcription factor 8 (PAX8), thyroid transcription factor 1 (TTF1), thyroid transcription factor 2 (TTF2), NKX2-5, and TSHR, play important roles during thyroid morphogenesis (4). Although mutations in these genes can lead to TD, its pathogenesis remains unclear.

Hematopoietically-expressed homeobox gene (HHEX), located on human chromosome 10q24, contains a 5.7 kb coding sequence divided into four exons (5,6) and encodes a homeodomain-containing transcription factor, first identified in multipotent hematopoietic cells. HHEX is expressed in the primordium of several organs derived from the foregut, including the thyroid bud (7). Studies of HHEX knock-out mouse strongly suggested that HHEX has no role in thyroid specification but is required to maintain Tif-1, Pax8, and Tif-2 expression in the developing thyroid (4). In this present study, we hypothesized that the HHEX possibly contributed to the development of TD in humans and aimed to identify potential pathogenic HHEX mutations in 234 Chinese children with TD, thereby providing insights into its etiology.

## METHODS

A total of 234 TD patients (94 boys, 140 girls, sex ratio 1:1.5, age 1.7±0.8 years), who were examined to make sure that they did not have other congenital anomalies such as congenital heart disease, congenital deafness, congenital cleft lip, congenital megacolon, were recruited through the neonatal screening program conducted in Qingdao, Yantai, Weifang, Jinan and Liaocheng in Shandong Province, China, from 2008 to 2012. Within the context of this same program, all measurements at five different laboratories in the relevant cities were done using the same assay. Neonatal screening for CH using filter paper was conducted in all of the subjects at 72 hours after birth. The blood samples were collected from the heel and TSH level was measured by enzyme-linked immunosorbent assay (ELISA). Subjects with increased TSH (TSH ≥20 uIU/mL) levels during this neonatal screening were invited for further evaluation. In these subjects, serum TSH (normal range 0.27-4.2 uIU/mL) and free thyroxine (fT4, normal range 12-22 pmol/L) were determined using electrochemiluminescence assay. The diagnosis of CH was based on a high serum TSH level and a low fT4 level. The diagnosis of TD was based on thyroid scintiscan or thyroid ultrasound examinations. Mutationsin PAX8, TTF1, and TTF2 in these patients were excludedin our previous studies (8). This present study was approved by the Ethics Committee of the Affiliated Hospital of Qingdao University. The blood samples from the children with TD were collected after written informed consent was obtained.

### Deoxyribonucleic Acid Analysis

Genomic deoxyribonucleic acid (DNA) was extracted from peripheral blood leukocytes using the phenol-chloroform method. The four exons and nearby introns in HHEX were amplified. Three pairs of specific primers were designed by PRIMER 5, polymerase chain reaction (PCR) was performed in 25 uL, using 250 nMdNTPs, 100 ng of template DNA, 0.5 uM of each forward and reverse primer, and 1.25 U AmpliTaq Gold DNA polymerase, in 1× reaction buffer (10 mMTrisHCl, pH 8.3, 50 mMKCl, 2.5 mM MgCl2). Samples were denatured at 94 oC for 5 minutes followed by 35 cycles of amplification. Each cycle consisted of denaturation at 95 oC for 30 seconds, at primer specific annealing temperature for 45 seconds, and primer extension at 72 oC for 45 seconds. After the last cycle, the samples were incubated for an additional 10 minutes at 4 oC to ensure that the final extension step was complete. The amplified products were analyzed in 1.5% agarose gel. In order to perform mutational analysis, amplified PCR products were purified and sequenced using the appropriate PCR primers and the DNA sequencing kit-BigDye Terminator Ready Reaction Cycle Sequencing Kit (PE Applied Biosystems, Warrington, UK) and run on an automated sequencer, ABI 3730XL (Applied Biosystems). The same region was sequenced in 168 blood samples from control individuals. All analyses were performed by statistical software package Statistical Package for the Social Sciences 19.0. Differences in the distribution of genotype and allele between case-control groups were analyzed by the chi-square method. The level of statistical significance was defined as a p-value <0.05.

## RESULTS

**Figure 1:**
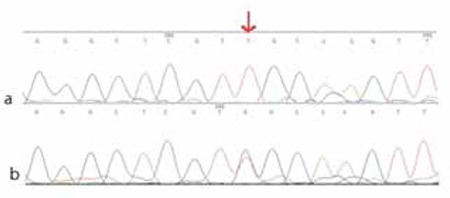
Partial sequences of HHEX. The variant is indicated by the arrow and it is 127 from splice site of intron 2.

Using thyroid scintiscan or thyroid ultrasound examinations, TD cases were divided into three groups according to the location and size of the thyroid gland, as agenesis (83 cases, 35.5%), ectopy (85 cases, 36.3%), and hypoplasia with normal location (66 cases, 28.2%) (Table 1). In all TD cases, the four exons and nearby introns of HHEX were amplified by PCR. The agarose gel electrophoresis indicated that the amplified products were in accordance with the target fragment we wanted. After direct sequencing, the sequences were analyzed using the Chromas and Sequencher and Nucleotide BLAST software programs. Sequence analysis of HHEX did not show any non-synonymous variance in the coding regions; however, we found a variant (rs2275729) which results in nucleotide T to G substitution (IVS2+127 G/T, 10.26%) in the intron 2 in 24 TD cases (Figure 1). These cases included 10 ectopy cases, 12 agenesis cases, and 2 hypoplasia cases. The frequency of this variant in the controls was 6.55%. Moreover, the differences in the allelic and genotypic frequencies between patients and controls were not statistically significant (c2=1.692, df=2, p=0.193 by genotype; c2=1.615, df=1, p=0.204 by allele) (Table 2).

## DISCUSSION

The thyroid follicular cells (TFCs), the most numerous cells of the thyroid gland that form the thyroid follicles, are spherical structures serving as a storage site for thyroid hormones and are essential for thyroid morphogenesis (9). The absence of TFCs in orthotopic or ectopic location leads to athyreosis. Lack of formation of the thyroid budor alterations in any of the steps following the differentiation of the thyroid bud such as defective survival and/or proliferation of the precursors of the TFCs can also cause this condition. The developing thyroid is unable to migrate to its definitive location anterior to the trachea, resulting in an ectopic thyroid gland. Up to now, only mutations in the thyroid transcription factors, such as PAX8, TTF1, NKX2-5, TTF2, and TSHR were associated with TD.

HHEX is a member of the homeobox family of transcription factors which play important roles in regulating the tissue-specific gene expression that is required for tissue differentiation, as well as in determining temporal and spatial patterns of development (10). HHEX has been shown to have a significant role in vertebrate thyroid development. In HHEX knock-outmouse embryos at E9, the thyroid primordium is present and this does not affect the expression of Ttf1, Pax8, and Ttf2. At E10, in the absence of HHEX, thyroid budding is severely impaired and the thyroid anlage is represented only by a few non-migrating cells which do not express Ttf1, Pax8, or Ttf2 messenger ribonucleic acid (mRNA). At later stages, the anlage disappears (4). These data strongly showed that HHEX is required for the development of the thyroid in the embryo. In addition, HHEX plays an important roleinzebrafish thyroid development. Elsalini et al (11) injected HHEX morpholino antisense RNA into one-cell-stage embryos and found that 82% of HHEX morphants with injection of 0.17 mM HHEX develop heart edema and have no thyroid follicles. With injection of lower concentrations of HHEX morpholino, a higher percentage of larvae were reported to show some follicle differentiation. Coinjection of HHEX mRNA restores follicle development in part of the morphants (11).

Further researchers suggested that the survival and growth of thyroid progenitors depend on a thyroid-specific signature of transcription factors. The combined expression of HHEX, Ttf2, Pax8, and Ttf1 is important for the development of thyroid (3), which is highlighted by their impact on regulation of thyroid-specific genes and functional differentiation of follicular cells. In thyroid progenitor cells, HHEX, Pax8, Ttf2, and Ttf1 form a regulatory network which probably occurs both at the level of promoter binding and by physical interaction with the other transcription factors (12). Previous studies demonstrated that the HHEX promoter is the binding and transactivating site of Ttf1 (13) and Pax8 (14). Pax8 interacts physically with Ttf1 (15), and the transcription of Ttf2 is activated by Pax8 (16). Besides, HHEX (13) and Ttf1 (16) regulate their own promoters automatically.

In this study, we screened potential causative mutations in HHEX in Chinese children with TD. However, we did not find any causative mutations in the HHEX coding regions. Al Tajialso found no HHEX mutations which were analyzedin a female hypothyroid patient (17). Interestingly, we found a variant which results in nucleotide T to G substitution (IVS2+ 127 G/T, 10.26%) in the intron 2 in TD cases. Although the intronic regions in which the variant located do not translate to amino acid sequences, they could potentially affect the protein product by changing the splicing site in RNA and therefore the final mRNA product. In other words, this part of human genome possesses significant regulatory elements that could affect gene expression. Due to its relatively distant location (IVS2+ 127 G/T) from splice site of intron 2, this variant may not effect on the splicing of the HHEX RNA. However, functional assessment of this variant will be needed in future studies. Our results also indicate that whole genome sequencing is a valuable tool for understanding variations in the human genome in our population.

To the best of our knowledge, this is the first attempt to examine the mutation in HHEX in a large sample of TD cases. The negative results of direct sequencing may have been caused by the limitations in our study. First, the causative mutation of HHEX for TD cases may not exist in the coding regions. Second, this is a highly selected population and sample size is relatively small in this study. Therefore, further studiesare still needed to determine the important role of HHEX during thyroid development, which may give an insight to the etiology of thyroid defects.

## Figures and Tables

**Table 1:**
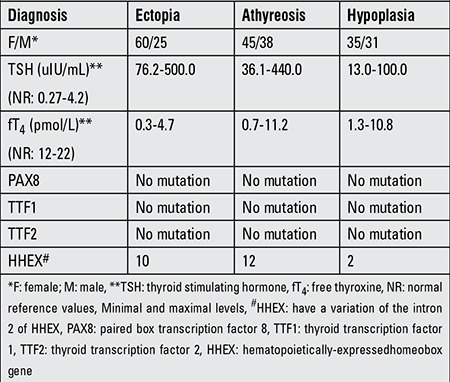
Clinical andlaboratoryfindings of patients with thyroid dysgenesis

**Table 2:**
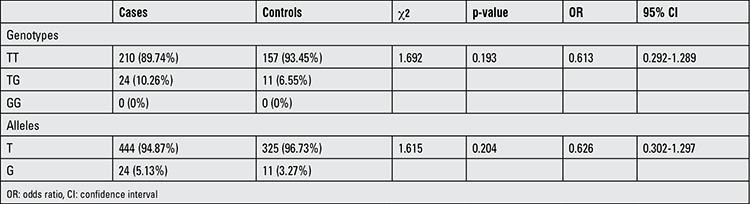
The genotypic and allelic frequencies of the HHEX IVS2+127 G/T (rs2275729) in the study and control groups

## References

[1] Cassio A, Corbetta C, Antonozzi I, Calaciura F, Caruso U, Cesaretti G, Gastaldi R, Medda E, Mosca F, Pasquini E, Salerno MC, Stoppioni V, Tonacchera M, Weber G, Olivieri A, Italian Society for Pediatric Endocrinology and Diabetology, Italian Society for the Study of Metabolic Diseases and Neonatal Screening, Italian National Institute of Health, Italian National Coordinating Group for Congenital H, Italian Thyroid Association, Italian Society of Pediatrics, Italian Society of Neonatology, Italian Society of Endocrinology, Associazione Medici Endocrinologi (2013). The Italian screening program for primary congenital hypothyroidism: actions to improve screening, diagnosis, follow-up, and surveillance. J Endocrinol Invest.

[2] Rastogi MV, LaFranchi SH (2010). Congenital hypothyroidism. Orphanet J Rare Dis.

[3] De Felice M, Lauro R (2004). Thyroid development and its disorders: genetics and molecular mechanisms. Endocr Rev.

[4] Nettore IC, Cacace V, De Fusco C, Colao A, Macchia PE (2013). The molecular causes of thyroid dysgenesis: a systematic review. J Endocrinol Invest.

[5] Morgutti M, Demori E, Pecile V, Amoroso A, Rustighi A, Manfioletti G (2001). Genomic organization and chromosome mapping of the human homeobox gene HHEX. Cytogenet Cell Genet.

[6] Crompton MR, Bartlett TJ, MacGregor AD, Manfioletti G, Buratti E, Giancotti V, Goodwin GH (1992). Identification of a novel vertebrate homeobox gene expressed in haematopoietic cells. Nucleic Acids Res.

[7] Thomas PQ, Brown A, Beddington RS (1998). Hex: a homeobox gene revealing peri-implantation asymmetry in the mouse embryo and an early transient marker of endothelial cell precursors. Development.

[8] Liu SG, Zhang SS, Zhang LQ, Li WJ, Zhang AQ, Lu KN, Wang MJ, Yan SL, Ma X (2012). Screening of PAX8 mutations in Chinese patients with congenital hypothyroidism. J Endocrinol Invest.

[9] Mauchamp J, Mirrione A, Alquier C, Andre F (1998). Follicle-like structure and polarized monolayer: role of the extracellular matrix on thyroid cell organization in primary culture. Biol Cell.

[10] Deng XP, Zhao LX, Wang BB, Wang J, Cheng LF, Cheng Z, Suo PS, Li H, Ma X (2013). The HHEX gene is not related to congenital heart disease in 296 Chinese patients. World J Pediatr.

[11] Elsalini OA, von Gartzen J, Cramer M, Rohr KB (2003). Zebrafish hhex, nk2.1a, and pax2.1 regulate thyroid growth and differentiation downstream of Nodal-dependent transcription factors. Dev Biol.

[12] Fagman H, Nilsson M (2010). Morphogenesis of the thyroid gland. Mol Cell Endocrinol.

[13] Puppin C, D’Elia AV, Pellizzari L, Russo D, Arturi F, Presta I, Filetti S, Bogue CW, Denson LA, Damante G (2003). Thyroid-specific transcription factors control Hex promoter activity. Nucleic Acids Res.

[14] Puppin C, Presta I, D’Elia AV, Tell G, Arturi F, Russo D, Filetti S, Damante G (2004). Functional interaction among thyroid-specific transcription factors: Pax8 regulates the activity of Hex promoter. Mol Cell Endocrinol.

[15] Palma T, Nitsch R, Mascia A, Nitsch L, Lauro R, Zannini M (2003). The paired domain-containing factor Pax8 and the homeodomain-containing factor TTF-1 directly interact and synergistically activate transcription. J Biol Chem.

[16] D’Andrea B, Iacone R, Palma T, Nitsch R, Baratta MG, Nitsch L, Lauro R, Zannini M (2006). Functional inactivation of the transcription factor Pax8 through oligomerization chain reaction. Mol Endocrinol.

[17] Al Taji E, Biebermann H, Límanová Z, Hníková O, Zikmund J, Dame C, Grüters A, Lebl J, Krude H (2007). Screening for mutations in transcription factors in a Czech cohort of 170 patients with congenital and early-onset hypothyroidism: identification of a novel PAX8 mutation in dominantly inherited early-onset non-autoimmune hypothyroidism. Eur J Endocrinol.

